# School Influences on Adolescent Depression: A 6-Year Longitudinal Study Amongst Catholic, Government and Independent Schools, in Victoria, Australia

**DOI:** 10.1007/s10943-022-01515-7

**Published:** 2022-03-14

**Authors:** Bosco C. Rowland, Mohammadreza Mohebbi, Adrian B. Kelly, Michelle L. Benstead, Jess A. Herde, Elizabeth M. Clancy, Jennifer A. Bailey, Bill Hallam, Paul Sharkey, Robyn Horner, John W. Toumbourou

**Affiliations:** 1 Centre of Social, Early and Emotional Development, School of Psychology, Faculty of Health, Deakin University, Geelong, VIC 3125 Australia; 2 Faculty of Health, Biostatistics Unit, Deakin University, Geelong, VIC Australia; 3 Queensland University of Technology, Brisbane City, QLD 4000 Australia; 4 School of Social Work, Faculty of Medicine, Dentistry and Health Sciences, The University of Melbourne, Parkville, VIC 3010 Australia; 5 Centre for Adolescent Health, Royal Children’s Hospital, Melbourne, VIC Australia; 6 Murdoch Children’s Research Institute, Melbourne, VIC Australia; 7 University of Washington, Seattle, WA 98115 USA; 8 Melbourne Archdiocese Catholic Schools, East Melbourne, VIC 3002 Australia; 9 Australian Catholic University, Fitzroy, VIC 3065 Australia

**Keywords:** Depression, Trajectories, Sector, Prosocial, Piecewise, Longitudinal

## Abstract

This study examined associations between school sector (Government, Catholic or Independent) and depressive symptomology over the secondary school years. Six waves of data collected annually from a representative Australian sample were examined. Multilevel piecewise linear and logistic regression controlling for a variety of demographic variables and protective factors was undertaken. In all sectors, depressive symptomology decreased between 10 and 13 years of age, but significantly increased for girls at age 13. Adolescents in Catholic schools reported significantly fewer symptoms of depression compared to those in Government and Independent schools. Adolescents in Catholic schools were less likely to report clinical levels of depressed mood compared to adolescents in Government schools.

**Supplementary Information:**

The online version contains supplementary material available at 10.1007/s10943-022-01515-7.

## Introduction

Poor mental health—defined as an individual’s reduced ability to cope with the normal stresses of living—is a major contributor to global disability (World Health Organisation, 2017). It is often associated with reduced quality of life, poverty, low productivity, social problems, drug and alcohol use, and early school leaving (Patton et al., 2016; World Health Organisation, 2017). The prevalence of mental health disorders rapidly increases during adolescence before peaking in early adulthood (Patton & Viner, 2007). With approximately 25% of the world population comprising adolescents (1.9 billion persons) (World Health Organisation, 2009), an increased understanding of how to prevent mental health problems emerging in adolescence is critical to international global health.

Puberty often defines the starting point for adolescence (World Health Organisation, 2001). While the chronological age for the onset of puberty has been declining in developed countries such as Australia, it has stabilised at approximately 12–13 years of age (Parent et al., 2003). Typified by a variety of physical, psychological, emotional, and hormonal changes, puberty has been linked with poor adolescent mental health (Hinchliff et al., 2016; Li et al., 2014). While similar levels of depression are reported for males and females in early adolescence, (Garrison et al., 1997) by the middle adolescent years (14–18) rates of depressive disorders in females are two-fold those of males (Wichstrøm, 1999). These gender differences persist well into early adulthood (Patton et al., 2008).

Depressive symptoms have been associated with higher levels of family conflict, difficulties in adjustment to secondary school, and peer victimisation (Chan et al., 2013; Resnick et al., 1997; Thomas et al., 2015). The quality of family relationships is linked to key indicators of adolescent mental health problems (Kelly et al., 2016) with early, compared to older, adolescents more vulnerable to alcohol use when there is a low emotional attachment to a parent (Kelly et al., 2011). Social Development Theory (Haggerty & McCowan, 2018), Attachment Theory (Eivers & Kelly, 2020), and Beneficial Action Theory (Toumbourou, 2016) suggest that the bonds individuals form with others within their socialising units (Mikulincer et al., 2005), and the opportunities, skills, and rewards/recognition arising as part of these social bonds, are critical to making meaning across the life course. Reviews and meta-analyses identify positive social bonds and the pro-social behaviour these bonds can engender as being protective against poor mental health (Blank et al., 2010; Coyne et al., 2018; Luberto et al., 2018; Teding van Berkhout & Malouff, 2016).

Schools are one setting where prosocial activities are promoted (Australian Curriculum Assessment and Reporting Authority (ACARA), 2020). In Australia, primary (5–12 years of age) and secondary (12–18 years of age) schools are organised into three sectors: Government, Independent and Catholic. Government schools are primarily funded by State Governments and grounded in legislation that promotes free, secular and compulsory education. Independent schools are often historically affiliated with a religious tradition (predominantly Christian), and while partially funded by Government, are also fee-paying. Catholic schools have a similar funding model to Independent schools but cover a broader demographic which includes many low SES schools. While all schools, regardless of sector, promote prosocial and civic engagement (Australian Curriculum Assessment and Reporting Authority (ACARA), 2020), religious and faith-based schools are often characterised by an overt ethos that explicitly promotes such behaviours in the school and broader community. For example, Catholic schools typically aim to develop an understanding of Christian texts and traditions that promote care, advocacy and action for the marginalised (Catholic Education Melbourne, 2020b), with curriculum and extra-curricula activities explicitly grounded in these principles (Catholic Education Melbourne, 2020a; Oxfam, 2020).

A recent meta-analysis of 93 studies suggested that religious contexts and settings may prime and promote prosocial attitudes and behaviours (Shariff et al., 2016). The strongest priming effects were found where there was active cognitive engagement with the culturally transmitted beliefs. Hence, it is possible that over and above the effects of engaging in school-based prosocial and civic activities, children in schools with an overt and explicit culture of service and commitment to social justice and prosocial activities may have better social and emotional outcomes, compared to children in schools that do not.

While there is a small number of studies examining sectoral differences in academic outcomes (e.g., Carbonaro & Covay, 2010; LePore & Warren, 1997), to the knowledge of the authors, there are no longitudinal studies exploring school sectorial differences and depressive symptomology, and the role of protective factors. The present study examined school-level sectoral differences in mental health symptoms for a representative sample of children in the Australian state of Victoria. We hypothesised that over the secondary school years, and while controlling for known protective factors, adolescents in schools characterised by an overt faith tradition and ethos (that is, those in Catholic and Independent schools) would have lower levels of depressive symptomology, compared to children in schools without an overt faith tradition.

## Method

### Design and Participants

Data was drawn from the International Youth Development Study (IYDS). The IYDS is an ongoing longitudinal study exploring the development of healthy and problematic behaviours in adolescents and young adults in Washington State (United States) and Victoria (Australia). The Victorian sample formed the analytic sample for the present study. Data collection began in 2002, with participants most recently surveyed in 2019–20 (aged 29–31). Data collected in the first six waves of the study (2002 through 2008) are analysed in the current study, as this covers the school years.

### Sampling Frame

The IYDS achieved a state-representative sample through the use of a two-stage cluster sampling approach. In stage one, Government, Catholic and Independent schools were stratified according to geographic location and schools were selected at random using a probability proportionate to grade-level size sampling procedure. In Stage Two, one class within each school was selected at random. At Wave 1 (2002), the Victorian sample comprised participants across three cohorts: Year 5 (youngest), Year 7 (middle) and Year 9 (oldest). At baseline, the average age in Year 5 was 11.0 (SD = 0.4), Year 7 was 13.0 (SD = 0.4) and Year 9 was 14.9 (SD = 0.4). Full details on sampling and recruitment have been previously published (McMorris et al., 2007). Key features of the Victorian cohort at Wave 1 are presented in Table 1.

**Table 1:** Demographics and Key Variables at Wave 1

		Wave 1
Youngest	*N*	927
	Year (grade)	5
	Age M (SD)	11.0 (0.4)
	Female	51.9%
	SES M (SD)	1.9 (0.4)
	SMFQ M (SD)	6.2 (4.9)
	SMFQ > 12	16.3%
	Puberty score	1.80
	Government	72.5%
	Independent	7.2%
	Catholic	20.3%
Middle	*N*	984
	Year (grade)	7
	Age M (SD)	13.0 (0.4)
	Female	50.8%
	SES M (SD)	1.9 (0.5)
	SMFQ M (SD)	7.1 (5.2)
	SMFQ > 12	22.9%
	Puberty Score M	2.31
	Government	62.8%
	Independent	15.4%
	Catholic	21.9%
Oldest	*N*	973
	Year (grade)	9
	Age M (SD)	14.9 (0.4)
	Female	52.2%
	SES M (SD)	1.9 (0.5)
	SMFQ M (SD)	7.8 (5.6)
	SMFQ > 12	26.3v %
	Puberty score	2.99
	Government	62.0%
	Independent	14.1%
	Catholic	24.0%
Total	*N*	2884
	Age M (SD)	13.0 (1.6)
	Female	51.9%
	SES M (SD)	1.9 (0.5)
	SMFQ M (SD)	7.0 (5.3)
	SMFQ > 12	21.9%
	Puberty score	2.38
	Government	65.6%
	Independent	12.3%
	Catholic	22.1%

*M* mean, *SD* standard deviation, *SMFQ* Short moods and feelings questionnaire score

### Procedure and Ethics

All IYDS (Victoria) procedures were approved by the Royal Children’s Hospital Ethics in Human Research Committee in Victoria and The University of Melbourne Human Ethics Committee. Permission to conduct research in schools was obtained from relevant state educational authorities. Active parent consent and student assent were obtained.

### Measures

The IYDS survey was adapted from the communities that care (CTC) Youth Survey (Arthur et al., 2002, 2007). The CTC Youth Survey assesses a range of risk and protective factors known to be associated with adolescent health and behaviour (e.g., delinquency, substance use). Measures have shown cross-sectional and longitudinal validity and reliability when administered in Victoria (Mason et al., 2011). Cronbach alphas (α) for all the scales at Wave 1 are presented below. Similar α scores for all scales were observed in all waves.

#### Dependent Measure

*Depressive symptoms* were measured using the 13-item Short Moods and Feelings Questionnaire (SMFQ: Angold et al., 1995). The questionnaire screens for symptoms of depression using a 3-point scale ranging from “Not true” (0) through to “True” (2). SMFQ total scores range from 0 to 26; scores were summed to produce a total SMFQ score and also recoded using the cut point of 12 which is indicative of depression (α = 0.87) (Turner et al., 2014). Scores of 7 are indicative of sub-clinical levels of depression.

#### Time

Given the rapidity of biopsychosocial change across the adolescent years, participants reported their exact age in years and months and this was used as the time variable in the modelling. The terms time and age are used interchangeably in the modelling description below.

#### Puberty

The Pubertal Development Scale (PDS) was used to assess the stage of puberty (Carskadon & Acebo, 1993). The scale comprises of five items: three questions common for both males and females (α, males = 0.65; females = 0.70). “Would you say that growth in height (growth spurt)?” “Would you say that body hair growth?” (responses were on a 4-point scale ranging from 1 “has not started” to “seems complete”).

Males were asked whether their voices were deepening, and whether they had begun to grow hair on their faces. Females completed items asking about whether their breasts had begun to grow and whether they had begun to menstruate. Responses were answered ‘Yes’ or ‘No’ and these responses were coded as 4 or 1, respectively, to align the magnitude of scores on this item to other items in this scale. The mean PDS score was used to operationalise overall pubertal development.

### Protective Factors Variables

*Protective factors* were organised into proximal versus distal influences: peer-individual factors, family factors and school factors were the proximal protective factors and community factors were the distal protective factors. Scales were scored, so higher scores indicated greater protection.

#### Peer-Individual Protective Factors

*Religiosity* was measured using the item “how often do you attend religious services or activities?” Responses: “Never” (1) through to “About once a week or more” (4). *Belief in Moral Order* was measured using four items (e.g., “I think it is okay to cheat at school”, α = 0.71). *Emotional Control* was measured using four questions (e.g., “I know how to calm down when I am feeling nervous”, α = 0.72). Both items were rated on a 4-point scale (*1* ‘NO!, *2* ‘no’, *3* ‘yes’, *4* ‘YES!). *Interaction with prosocial peers* was measured using two items (e.g., “In the past 12 months, how many of your friends tried to do well in school?”, α = 0.32), with responses ranging from 1 “None of my friends” through to 5 “4 of my friends” *Rewards for Prosocial Involvement* was measured with two items (e.g., what are the chances you would be seen cool if you were involved with clubs, α = 0.40), with responses ranging 1: “No or Very Little Chance” to 5 “Very Good Chance”.

#### Family Protective Factors

*Attachment to parents* was measured using four items (e.g., “Do you feel close to your mother?”, α = 0.76). *Opportunities for Prosocial Involvement* were measured using three items (e.g., “My parents ask me what I think before most family decisions affecting me are made?”, α = 0.73). Responses were made on a 4-point scale (*1* ‘NO!, *2* ‘no’, *3* ‘yes’, *4* ‘YES!). *Rewards for Prosocial Involvement were measured with* two items (e.g., “My parents notice when I am doing a good job and let me know about it (α = 0.75). Responses: “Never or Almost Never” (1) through to “ALL the Time” (4).

#### School Protective Factors

*Opportunities for Prosocial Involvement* were measured using 5-items relating to activities at school (e.g., “I have lots of chances to be part of class discussions and activities” (α = 0.60). *Rewards for Prosocial Involvement* were measured using four items (e.g., ‘The school lets my parents know when I have done something well’) (α = 0.71). Items for both scales were rated on a 4-point scale ranging from *1* ‘Never or Almost Never’ to *4* ‘ALL the Time’.

#### Community Protective Factors

*Opportunities for Prosocial Involvement* were measured using four items (e.g., involvement in Scouts, Brownies; youth clubs (α = 0.66). *Rewards for Prosocial Involvement* were measured with three items (e.g., “My neighbours notice when I do a good job… “α = 0.87). Responses for both scales were made on a 4-point Likert scale (*1* ‘NO!, *2* ‘no’, *3* ‘yes’, *4* ‘YES!).

### Demographics

Family socio-economic status (SES) was based on parent report for the highest level of education (e.g., completed secondary school) and level of family income in 2002 (ranging from < $10 K to 200 K +). Participants reported gender as either male or female.

### Analytical Approach

To test the hypothesis, multilevel growth models (MGM) were conducted using Stata, version 17. Two main models were developed: (1) A piecewise regression model (Mitchell, 2012) which examined the potential non-linearity association of age with total scores of the SMFQ as a continuous measure; (2) A binary regression model that used the clinical cut-point of 12 for the SMFQ. Two additional models are included in the online material: one where the dependent variable (DV) is a continuous measure of SMF (i.e., not piecewise), and another, binary model where the dependent variable is a subclinical cut-point of 7 on the SMF.

Piecewise models are sometimes referred to as spline or broken stick models as they can be used to account for nonlinearity between a predictor and an outcome of interest. They do this by fitting separate lines segments demarcated by what are referred to as knots or jumps. Knots are where the regression line changes its slope. A jump is when there is a sudden change in the magnitude of the dependent variable—the slope may remain the same, or it may change. This study examined a change in slope direction and magnitude (jump) of depressive symptomology at the onset of a common age of puberty (13 years).

For this study, knots were created using the mkspline command in Stata. This command created a variable representing time before the knot (age 13) and a variable representing a time after the knot (see T1 and T2 in Table 3). To model a possible significant change in depressive symptoms at age 13, a binary variable representing age 13 or greater was incorporated into the model. More detail about the mkspline command in Stata and how to use dummy variables to represent jumps at particular time points in piecewise modelling is available in Mitchell (2012).

### Model Building Approach

Following Singer and Willett (2003), five steps were used to develop the MGMs: In step one, an intercept-only model was examined—an unconditional mean (null). This model represents the average intercept (starting SMF score at age 10, without confounder adjustment), for all participants. The second step utilised an unconditional growth model, where the age variable (representing time) was the only variable added to the model. This model showed the effect of age on the dependent variable, without adjusting for confounders. As described above, for the piecewise model, there were two-time variables (T1 & T2): T1 represented ages between 10 and 13, where 13 represented the average age of puberty onset; T2 represented ages 13–18 (see Table 2, below). The binary variable representing a substantial jump at age 13 was also included in the model.

**Table 2:** Null, unconditional growth, and sectoral models for piecewise and binary regression

Piecewise	Unconditional mean (Null)		Unconditional growth		Sectorial	
			Coefficient	95 CI	Coefficient	95 CI
T1			−0.21	[−0.54, 1.57]	−0.20	[−0.53, 0.14]***
T2			0.39	[0.30, 0.48]***	0.42	[0.32, 0.51]
Intercept 13			1.00	[0.43, 1.62]**	0.97	[0.41, 1.54]
Sector: Government					Referent	
Independent					−0.41	[−0.87, 0.05]
Catholic					−0.41	[−0.79, −0.02]*
Constant	7.36	[7.18, 7.53]***	5.67	[5.37, 5.96]***	5.74	[5.43, 6.05]***
var(age)	N/A		0.69	[0.58, 0.82]	0.70	[0.59, 0.84]
variance (constant)	16.83	[15.62, 18.13]	14.16	[11.91, 16.84]	13.61	[11.38, 16.27]
variance (residual)	20.71	[20.03, 21.42]	17.36	[16.73, 18.01]	17.20	[16.57, 17.86]
Covariacnce (age & constant)	N/A		−1.05	[−1.51, −0.59]	−1.01	[−1.48, −0.55]
*N*	9730		9723		8729	
Individuals	2880		2880		2879	
df	3		6		8	
Log likelihood	−30,371		−30,068		−26,349	
AIC	60,749		60,147		52,773	
BIC	60,770		60,190		53,042	

	Unconditional Mean (Null)		Unconditional Growth		Sectorial	
Binary cutpoint 12	OR	95 CI	OR	95 CI	OR	95 CI
Age_c			1.08	[1.20, 1.14]***	1.08	[1.02, 1.34**
Sector: Government					Referrent	
Independent					0.72	[0.56, 0.93]
Catholic					0.73	[0.59, 0.90]
Constant	0.21	[0.19, 0.23]***	0.16	[0.14, 0.19]***	0.19	[0.16, 0.21]***
var(age)	N/A		0.09	[0.06, 0.14]	0.09	[0.06, 0.15]
variance (constant)	2.54	[2.19, 2.95]	3.21	[2.75, 3.74]	3.17	[2.71, 3.71]
variance (residual)	N/A		0.29	[0.14, 0.44]***	0.32	[0.17, 0.47]***
*N*	10,235		10,004		9,	
Individuals	2884		2884		2884	
df	2		4		7	
Log likelihood	−5361		−4977		−4885	
AIC	10,727		9965		9785	
BIC	10,741		10,001		9835	

95CI: 95 percent confidence interval; OR = Odds ratio; *p < ..05; **p < .01; ***p < .001; T1 = time coefficient between ages 10 and 13; T2 = time coefficient between ages 14 and 18; Intercept13 = intercept at age 13

*AIC* Akaike information criteria; *Age_c* Age grand mean centred; *BIC* Bayesian information criteria; *LL* log-likelihood; individuals = number of individual trajectories/cases in analyses; *N* number of data points

Because *sector* was the variable of primary interest, this was entered as a third step and retained throughout the model building process. This allowed an examination of whether the sector was associated with the SMF scores, before adjusting for confounders. A sector interaction with the two time variables was examined to assess how this association changed over the two-time periods (10–13 years and 13–18 years). The fourth step was the entry of the demographic variables (e.g., family SES and participant’s baseline age, pubertal status), which were retained throughout the model building and only removed in the final model if not significant. Because levels of depressed mood differ for males and females after puberty, gender interactions with puberty and gender interactions time were examined. The fifth step was the entry of protective factors, beginning with the most proximal and finishing with those most distal. All protective factors were grand mean centred.

## Results

Demographic variables at baseline are presented in Table 1. By cohort, descriptive statistics for key variables used in the analysis at Wave 1 are presented in Table S1, online supplementary material. For each wave, average SMFQ scores ranged between 7 and 8, with a standard deviation between 5 and 6. For each cohort, the mean score of each protective factor was approximately three, with standard deviations ranging between 0.4 and 1.

Table 2 contains the unconditional mean, unconditional growth, and school sectoral model for the piecewise model. The unconditional mean model (Step 1) indicated that the first intercept (age 10) was 7.36 (see constant in Table 2), suggesting on average for each participant of mild to moderate depression (sub clinical) over the secondary school years. The Unconditional growth model (Step 2) which modelled time years 10 to 13 and also years 13 to 18, indicated that the negative association between age and SMFQ scores between ages 10 and 13 was not significant (*T*2 = −0.21; *p* > 0.05). However, there was a significant positive association with age after 13 years and SMFQ (*T*2 = 0.39; *p* < 0.000).

The variable used to model a possible jump (int) indicated that at age 13, there was a significant increase in SMFQ scores (int13 = 1.0; *p* < 0.001). The sectoral model (Step 3) indicated there was a significant association between the school sector and SMFQ scores. The unadjusted sectorial piecewise model indicated that SMF scores for Catholic schools were significantly lower than Government schools (β = 0.41; 95CI: −0.79 to 0.02 *p* < *0.05).* The unadjusted difference between Independent and Government schools was not significant (β = 0.41; 95%CI: −0.87–0.05; *p* =  > *0.05)* Pairwise comparisons indicated that the unadjusted difference between Catholic and Independent schools was not significant (0.01; *p* = *0.984*).

Table 3 presents the final adjusted piecewise growth model, using an SMF continuous score as the DV. Controlling for demographic factors (Step 4) and protective factors (Step 5), the school sector was a significant predictor of SMFQ scores. Adolescents in Catholic schools reported significantly lower levels of SMFQ scores compared to adolescents in Government schools (β = −0.39; *p* < 0.05). Adolescent SMF reports in independent schools were not significantly different from those in Government schools (0.18; 95%CI: −0.29 to 0.64; *p* > 0.05). Pairwise comparisons indicated students in Catholic schools reported significantly lower levels of depression, compared to those in Independent schools (−0.57; 95CI: −1.08, −0.05; *p* < 0.05).

**Table 3:** Final growth models for piecewise and binary cut-point 12 regression

Variable	Final Model−SMF Piecewise		Final Model SMF cutpoint 12		
	Coefficient	95CI	Variable	OR	95 CI
T1	−2.01	[−3.07, −0.95]***	Age (time)	0.95	[0.89, 1.02]
T2	−0.09	[−0.24, 0.05]			
Intercept 13	1.29	[0.17, 2.42]*			
*Sector*			*Sector*		
Government	Referent		Government	Referent	
Independent	0.18	[−0.29, 0.64]	Independent	0.99	[0.74, 1.33]
Catholic	−0.39	[−0.76, −0.02]*	Catholic	0.77	[0.61, 0.98]*
Age at baseline	−0.11	[−0.22, −0.01]*	Age at baseline	0.96	[0.90, 0.97]*
Socio economic status	−0.45	[−0.79, −0.10]*	Socio economic status	N/A	
Female	−14.45	[−22.18, −6.72]***	Female	2.93	[2.36, 3.63]***
Female#T1	1.25	[0.64, 1.86]***			
Female#T2	0.35	[0.16, 0.53]***			
Puberty	0.26	[0.00, 0.52]*	Puberty	0.88	[0.70, 1.10]
Female #puberty	N/A		Puberty#sex	1.67	[1.27, 2.18]***
P: rewards for prosocial	−0.15	[−0.29, 0.00]*	P: rewards for prosocial	0.90	[0.82, 0.99]***
P: peers invovled in prosocial	−0.16	[−0.29, −0.02]*	P: peers invovled in prosocial	0.89	[0.82, 0.97]*
P: belief in a moral order	−0.98	−1.21, −0.74*	P: belief in a moral order	0.59	[0.51, 0.69]***
P:emotional control	−1.96	[−2.16, −1.76]***	P:emotional control	0.38	[0.33, 0.43]***
F:rewards for prosocial involvement	−0.57	[−0.88, −0.26]***	F:rewards for prosocial involvement	0.76	[0.62, 0.92]**
F:rewards for prosocial involvement	−0.46	[−0.73, −0.20]***	F:rewards for prosocial involvement	0.78	[0.66, 0.92]***
F: opportunities for prosocial	−0.80	[0.01, −0.54]	F: opportunities for prosocial	0.66	[0.56, 0.77]***
S:opportunities for prosocial	0.36	[0.01, 0.71]			
S:rewards for prosocial	−1.00	[−1.29, −0.72]	S:rewards for prosocial	0.68	[0.58, 0.79]***
C:rewards for prosocial	0.20	[0.05, 0.34]**			
Constant	32.48	[19.75, 45.22]***	Constant	0.40	[0.18,0.87]***
Random effects (individual id)			Random effects (individual id)		
Variance (age)	0.63	[0.49, 0.81]	Variance (age)	0.08	[0.03, 0.22]
Variance (cons)	107.64	[80.18, 144.49]	Variance (cons)	2.59	[2.10, 3.18]
Variance (residual)	15.53	[14.86, 16.23]			
Covariance age#cons	−7.88	[−10.10, −5.66]	Covariance age#cons	−0.01	[−0.16, 0.15]
*N*	7821		*N*	8004	
Individuals	2713		Individuals	2725	
df	26		df	19	
Log-likelihood	−23,588		Log-likelihood	−3581	
AIC	47,228		AIC	7200	
BIC	47,409		BIC	7333	

A1 = slope 11–13 years; A2 = slope 13–18 years; Int13 = intercept at age 13; 95CI: 95 percent confidence interval; *P..05; ** *P*,.01; ***P,.001; *P* peer/individual protective factor; *F* family protective factor; S = School protective factor; *C* community protective factor; *pp* interaction with prosocial peers; *mo* belief in a moral order; *ec* emotional control; *rp* rewards for prosocial involvement; *fa* family attachment; *op* family opportunities for prosocial involvement; *rp* school rewards for prosocial involvement; # = interaction; All sector interactions has Government schools as the referent. *95CI* 95percent confidence interval; *OR* = odds ratio *p < 0.05; **p < 0.01; ***p < 0.001; P = peer/individual protective factor; F = family protective factor; S = School protective factor; # = interaction; df = degrees of freedom; AIC = Akaike information criteria, BIC = Bayesian information criteria; individuals = number of individual trajectories/cases in analyse

**Fig. 1:**
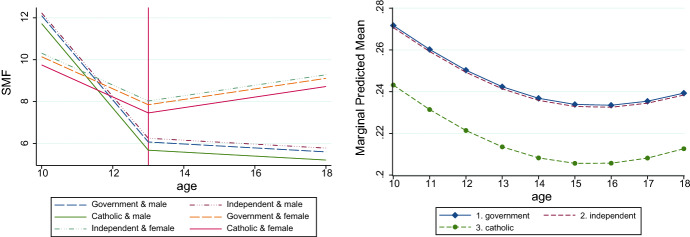
Predicted Marginal Effects Piecewise (left panel) and Binary Regression model (right panel). *Note: Girls are the top 3 lines; boys are the bottom 3 lines

A gender-by-puberty interaction was not identified. However, a significant gender-by-time interaction was identified. The gender time interaction was significant between the ages of 10–13 (female#T1 = 1.25; *p* < 0.001), and between the ages of 14–18 (female#T2 = 0.35; *p* < 0.001). This indicated females reported having an SMF score of 1.25 more than males between ages 10–13 and an SMF score of 0.35 more than males between ages 13 and 18. Figure 1 (left panel) presents marginal predictions for the final piecewise model. The figure shows the sectoral and gender differences in SMFQ scores over the six waves.

Most protective factors were retained in the final model. There were mostly negative associations between all protective factors and SMFQ scores; as protective factor scores increased, SMF scores decreased. However, the school factor “opportunities for prosocial involvement” (β = 0.36; *p* < 0.05), and the community factor of “rewards for prosocial involvement” (β = 0.20; *p* < 0.05) were associated with increases in SMF scores. Only two protective factors were removed from the final model—religiosity and community opportunities for prosocial involvement. The extent to which an adolescent had completed puberty was associated with increased levels of depression (β = 0.26; *p* < 0.05). Results for the standard regression model—using SMFQ as a continuous dependent variable—are presented in the online material (See Tables S2 & S3).

Table 2 contains the unconditional mean, unconditional growth, and unadjusted sectoral model for the binary model. The final binary model is presented in Table 3. The unconditional model indicated that without adjusting for confounders and time there was a 79% (Constant OR = 0.21; *p* < 0.001) chance that adolescents would not report SMF to score indicative of depressed mood (i.e., SMF > 12). The unconditional growth model indicated that not accounting for confounders, for every one year’s increase in age, the odds of reporting having symptoms scoring greater than 12 increased by 8% (OR = 1.08 (95CI: 1.02, 1.14); *p* < 0.001).

The sectoral model indicated that when controlling only for a time, adolescents in Catholic schools were 27% less likely to report having symptoms indicating the presence of depression, compared to adolescents in Government schools (OR = 0.73; 95CI: 0.56–0.90; *p* < 0.001). Similarly, adolescents in Independent schools were 28% less likely to report depressed moods, compared to adolescents in Government schools. (OR = 0.72; 95%CI: 0.56–0.93; *p* < 0.05). Pairwise comparisons indicated that the difference between adolescents in Catholic and Independent schools, in the odds of reporting depressive symptoms greater than 12, was not statistically significant (0.001, *p* > 0.05).

In the final binary model, controlling for demographic and protective factors, the school sector was a significant predictor of a clinical level of depressive symptomology (e.g., SMFQ > 12). Adolescents in Catholic schools were 23% less likely to report having clinical levels of depression compared to adolescents in Government schools (OR = 0.77; 95CI; 0.61–0.98; *p* < 0.05). The odds ratio for comparing clinical levels of symptoms between Independent and Government schools was not significantly different (OR = 0.99; 95CI; 0.74–1.33; *p* > 0.05). Pairwise comparisons indicated the difference in SMFQ scores between adolescents in Catholic and Independent schools was also not significantly different (OR = 0.77; 95CI: 0.56–1.07; *p* > 05).

Time was not a significant predictor of clinical levels of depressive symptoms. An interaction between gender and time was not identified. However, an identification was made between gender and puberty. Females who were more pubertally developed were 67% more likely to report clinical levels of depressive symptomology (OR = 1.67; *p* < 0.001), compared to males at a similar level of pubertal development. All protective factors retained in the final model were associated with a lower likelihood of reporting depression. Fewer protective factors were associated with reporting clinical levels of depressive symptomology (*n* = 8), compared to the model examining overall SMFQ scores (*n* = 10).

Figure 1 (right panel) presents marginal trajectory predictions for the final binary model. Marginal predictions are very similar for Independent and Government schools. The figure appears to show a visual difference between Catholic and Government schools, however, as indicated above, the pairwise comparisons were not significantly different. Comparable results are reported for the model with a subclinical cut point of 7 (see online material Tables S2 & S3).

## Discussion

Using data spanning 6 years, 3 cohorts and a State-representative Australian sample, this study examined the hypothesis that there would be school sectoral differences in reported levels of adolescent depressive symptomology over the secondary school years. Results from piecewise regression and logistic regression growth modelling partially supported this hypothesis. Specifically, over the high school years, while controlling for a variety of variables, adolescents in Catholic schools in Victoria reported significantly fewer symptoms than adolescents in Government schools and adolescents in Independent schools. When comparing clinical levels of symptomology, adolescents in Catholic schools were significantly less likely to report clinical levels of depressed mood compared to adolescents in Government schools. Significant differences in clinical levels of symptoms were not observed between other sectors (e.g., Catholic schools & Independent schools; and Independent & Government Schools).

Why would adolescents attending Catholic schools have lower levels of overall depressive symptomology than adolescents in other sectors? It is possible that it is related to the overt tradition and practices associated with the Catholic religious tradition. Prior research shows that when caring behaviour is intrinsically motivated and there are explicit, overt cognitive associations made, meaning and purpose are developed (Han, 2015). This may be associated with improved mental health (Heisel & Flett, 2016; Landstedt et al., 2016; Zechmann & Paul, 2019). Frankl (1988) argued that meaning and purpose are found in caring values and actions beyond self-interest. Similarly, Erikson (1968) argued that human maturity and mental health were developed by promoting care for the next generation. It could be that over and above the effects of engaging in school-based prosocial and civic activities, children in schools with an overt and explicit culture of service and commitment to social justice and prosocial activities, with a caring existential framework are the differentiating factor.

That religiosity was not a significant predictor may suggest that religiosity is possibly not related to depression symptomology. However, the measure of religiosity used was limited to one dimension (i.e., measures of church attendance) of many possible aspects of religiosity (Koenig et al., 2015). A multidimensional measure of religiosity may find an association with lower levels of symptomology. This suggestion is supported by the finding that belief in a moral order had a significant protective effect on depressive symptomology in all models (including those in the supplementary material). The measure of belief in a moral order indicates a personal belief in principles such as fairness, justice and charity, practices promoted in many religious traditions.

Adolescents in Catholic schools reporting fewer symptoms may also be associated more broadly with evidence-based and progressive, prosocially-oriented programs that promote positive mental health. The Catholic school system in Victoria during the course of the current study was an early adopter of evidence-based mental health promotion interventions, including the Gatehouse project (Patton et al., 2006) and the Resilient Families Project (Singh et al., 2019). Catholic schools also belong to a larger religious tradition which may foster a sense of support and belong in adolescent students in this sector.

While sector was a predictor of mental health, a variety of protective factors at the family level were associated with lower levels of depressive symptoms. These included having a close relationship with a mother or father. This is consistent with previous research (Webster-Stratton & Herman, 2008; Webster-Stratton et al., 2008). Family relationships present opportunities to learn to care beyond self-interest, through encountering the necessary personal costs attached to intrinsically motivated caring actions (Annas, 2004), These costs might include effort, courage, patience, commitment, and persistence, virtues often necessary to the undertaking of caring and prosocial behaviour (Baumeister et al., 2013; Blasi, 2005; Curzer, 2002). Teaching children to develop these social and prosocial skills can also help them to manage and prevent anxiety and depressive symptoms.

A variety of individual/peer factors were identified as a protective. These included: emotional control, interaction with prosocial peers, and peer rewards for prosocial involvement. Peer relationships are an important part of the development, as they teach children and adolescents to empathise with others (Domitrovich et al., 2007). Children also learn social and self-regulatory skills so that their peers do not reject them (Kam et al., 2004). The findings emphasise the importance for adolescents of identifying, understanding and regulating their emotions, and being able to take another person’s perspective. These skills are not only basic building blocks to development but also to the development of a civil and more caring society, while at the same time improving wellbeing.

School opportunities to engage in prosocial activity and community recognition for engagement in prosocial activities were significantly associated with increased depressive symptomology. This is consistent with previous studies and Social Determination Theory. When opportunities for prosocial action are promoted through routine practices, programs or curricula in a school or community (i.e., they are implemented from the top down), intrinsic motivation for prosocial action is often diminished. This is usually due to participants feeling they are participating to conform to some other ‘external motivation’ rather than it being a true expression of their own will or ‘intrinsic motivation’ (Grant, 2008). Having external demands and expectations placed on children sometimes has the effect of reducing independence and wellbeing (Gagne, 2003).

As found in other studies, girls were more likely to report a greater depressed mood than boys across puberty (Patton & Viner, 2007; Wichstrøm, 1999). The finding that girls are vulnerable to depressed moods across pubertal transitions highlights the critical opportunity of early adolescence as a window for the promotion of better mental health outcomes. Building awareness of the links between puberty and depressed mood, and strengthening resilience through the provision of evidence-based programs that are prosocially oriented and delivered prior to puberty may be key ways of better preparing early adolescents for the challenges of adolescence.

This study has a number of strengths and limitations. The retention rate and a variety of control variables used are strengths of the study. However, the findings are limited to the period and cohorts analysed (2002–2008) and may no longer apply to contemporary students. Catholic school culture sometimes differs between States and within State or Diocesan jurisdictions. To this end, the findings should be replicated with students in other Australian states and territories and other countries (e.g., IYDS: USA, India, Netherlands). As the cohorts used in this study are still being followed, the degree of symptomology sustained into adulthood should be examined. The majority of Independent schools in this study were classified as having a religious tradition; however, not all these Independent schools continue to do so, and some have distanced themselves from their founding tradition with a change of name. While the study is longitudinal, findings cannot be argued to be causal. Further examination should be given to why time (age) was not associated with clinical levels of depressed mood, but the age at baseline was associated with clinical levels of depressed mood.

## Conclusion

This is the first Australian study using a state-representative sample and longitudinal study design to examine associations between school sectors and depressive symptoms. Findings suggest that involvement in prosocial activities is associated with lower levels of depressed mood. There is evidence suggesting that where culture in schools is linked to curricula with philosophical and existential underpinnings of care and prosocial behaviour, this may protect adolescents from declines in mental health across the secondary school years.

## Supplementary Information

Below is the link to the electronic supplementary material.

Supplementary file1 (DOC 38 kb)

## Data Availability

All data and materials as well as software applications or custom codes used in this manuscript support their published claims and comply with field standards.

## Code availability

The statistical syntax/code used in the analysis for this publication is available on request.
