# Correction to: School Influences on Adolescent Depression: A 6-Year Longitudinal Study Amongst Catholic, Government and Independent Schools, in Victoria, Australia

**DOI:** 10.1007/s10943-022-01551-3

**Published:** 2022-03-30

**Authors:** Bosco C. Rowland, Mohammadreza Mohebbi, Adrian B. Kelly, Michelle L. Benstead, Jess A. Herde, Elizabeth M. Clancy, Jennifer A. Bailey, Bill Hallam, Paul Sharkey, Robyn Horner, John W. Toumbourou

**Affiliations:** 1 Centre of Social, Early and Emotional Development, School of Psychology, Faculty of Health, Deakin University, Geelong, VIC 3125 Australia; 2 Faculty of Health, Biostatistics Unit, Deakin University, Geelong, VIC Australia; 3 Queensland University of Technology, Brisbane City, QLD 4000 Australia; 4 School of Social Work, Faculty of Medicine, Dentistry and Health Sciences, The University of Melbourne, Parkville, VIC 3010 Australia; 5 Centre for Adolescent Health, Royal Children’s Hospital, Melbourne, VIC Australia; 6 Murdoch Children’s Research Institute, Melbourne, VIC Australia; 7 University of Washington, Seattle, WA 98115 USA; 8 Melbourne Archdiocese Catholic Schools, East Melbourne, VIC 3002 Australia; 9 Australian Catholic University, Fitzroy, VIC 3065 Australia

## Correction to: Journal of Religion and Health 10.1007/s10943-022-01515-7

In the original publication of the article, the Authors' Contributions statement were incorrectly published with X, Y, Z instead of the authors' names. This has been corrected with this erratum.

Authors’ contributions: All authors contributed to the manuscript conception and design. Material preparation, data collection were performed by Michelle Benstead, Elizabeth Clancy and Jess Heerde. The primary analysis was undertaken by Bosco Rowland and supported by Mohammadreza Mohebbi. The first draft of the manuscript was written by Bosco Rowland and all authors commented on previous versions of the manuscript. All authors read and approved the final manuscript. John Toumbourou and Jennifer Bailey were involved with securing the funds for data collection of the IYDS component of the study. Bosco Rowland, Robyn Horner and Paul Sharkey were involved with securing funding from the Melbourne Archdiocese of Catholic Schools (MACS).

The original article has been corrected.

