# Supplementary material for: School Influences on Adolescent Depression: A 6-Year Longitudinal Study Amongst Catholic, Government and Independent Schools, in Victoria, Australia
Source: J Relig Health. 2022 Mar 14;62(2):1136–56. doi: 10.1007/s10943-022-01515-7 (PMC10042755; doi:10.1007/s10943-022-01515-7)
Supplement: Supplementary file 1 — Supplementary file1 (DOC 38 kb) [file 10943_2022_1515_MOESM1_ESM.doc]

*Table S1 Distribution and Frequency of Control Variables Used in the Analysis at Wave 1*

|  | **Variable** | ***M (SD)*** | **Variable** | ***M (SD)*** |
| --- | --- | --- | --- | --- |
| **Youngest** | Religiosity | 2.3 (1.1) | Family rewards for prosocial involvement | 3.4 (0.5) |
|  | Belief in moral order | 3.6 (0.5) | School opportunities for prosocial | 3.2 (0.4) |
|  | Interaction prosocial peers | 3.3 (0.9) | School rewards for prosocial involvement | 3.2 (0.5) |
|  | Rewards for prosocial involvement | 3.5 (1.1) | Community opportunities for prosocial | 3.0 (0.7) |
|  | Emotional control | N/A | Community rewards for prosocial involvement | 2.6 (1.0) |
|  | Family attachment | 3.3 (0.6) | Pubertal Development | 1.8 (0.4) |
|  | Family opportunities for prosocial | 3.4 (0.6) |  |  |
| **Middle** | Religiosity | 2.1 (1.0) | Family rewards for prosocial involvement | 3.3 (0.6) |
|  | Belief in moral order | 3.3 (0.6) | School opportunities for prosocial | 3.1 (0.4) |
|  | Interaction prosocial peers | 3.2 (0.8) | School rewards for prosocial involvement | 3.0 (0.6) |
|  | Rewards for prosocial involvement | 3.3 (0.9) | Community opportunities for prosocial | 2.9 (0.7) |
|  | Emotional control | 2.7 (0.7) | Community rewards for prosocial involvement | 2.4 (0.9) |
|  | Family attachment | 3.1 (0.7) | Pubertal Development | 2.3 (0.6) |
|  | Family opportunities for prosocial | 3.2 (0.7) |  |  |
| **Oldest** | Religiosity | 2.0 (1.0) | Family rewards for prosocial involvement | 3.0 (0.6) |
|  | Belief in moral order | 3.0 (0.6) | School opportunities for prosocial | 2.9 (0.4) |
|  | Interaction prosocial peers | 3.0 (1.0) | School rewards for prosocial involvement | 2.8 (0.5) |
|  | Rewards for prosocial involvement | 3.1 (0.8) | Community opportunities for prosocial | 2.9 (0.7) |
|  | Emotional control | 2.6 (0.6) | Community rewards for prosocial involvement | 2.3 (0.9) |
|  | Family attachment | 2.9 (0.7) | Pubertal Development | 3.0 (0.5) |
|  | Family opportunities for prosocial | 3.0 (0.7) |  |  |
| **Total** | Religiosity | 2.1 (1.1) | Family rewards for prosocial involvement | 3.2 (0.6) |
|  | Belief in moral order | 3.3 (0.6) | School opportunities for prosocial | 3.0 (0.5) |
|  | Interaction prosocial peers | 3.2 (0.9) | School rewards for prosocial involvement | 3.0 (0.6) |
|  | Rewards for prosocial involvement | 3.3 (0.9) | Community opportunities for prosocial | 2.9 (0.7) |
|  | Emotional control | 2.6 (0.6) | Community rewards for prosocial involvement | 2.4 (0.9) |
|  | Family attachment | 3.1 (0.7) | Pubertal Development | 2.4 (0.7) |
|  | Family opportunities for prosocial | 3.2 (0.7) |  |  |

Table S**2 *Null, Unconditional Growth and Sectorial Models for SMFQ Continuous and Cut-point 7*

Notes: 95CI: 95 percent confidence interval; OR= odds ratio; **p* <.05; ** *p* <.01; ****p* <.001; Age_10 = age centred at 10. Thus x intercept at zero = age 10; Age_c = age centred at mean. AIC = Akaike information criteria, BIC= Bayesian information criteria; individuals= number of individual trajectories/cases in analyses.

Table S3 *Final Models for SMFQ Continuous and Cut-point 7*

Notes: 95CI: 95 percent confidence interval; OR = odds ratio **p* <.05; ***p* <.01; ****p* <.001; P= peer/individual protective factor; F= family protective factor; S= School protective factor; pp: interaction with prosocial peers; rp: rewards for prosocial involvement; fa: family attachment; op: family opportunities for prosocial = opportunities for prosocial involvement; # = interaction; df = degrees of freedom; AIC = Akaike information criteria, BIC = Bayesian information criteria; LL = log likelihood; individuals = number of individual trajectories/cases in analyses Age_10 = time variable (age centred at 10). Thus x-axis intercept at zero = age 10; Age = age centred at mean.
